# Phase Transformation in Tantalum under Extreme Laser Deformation

**DOI:** 10.1038/srep15064

**Published:** 2015-10-19

**Authors:** C.-H. Lu, E. N. Hahn, B. A. Remington, B. R. Maddox, E. M. Bringa, M. A. Meyers

**Affiliations:** 1 University of California, San Diego, La Jolla, CA, 92093, USA; 2 Lawrence Livermore National Laboratory, Livermore, CA, 94550, USA; 3 Facultad de Ciencias Exactas y Naturales, Universidad Nacional de Cuyo, Mendoza 5500, Argentina; 4 CONICET, Mendoza 5500, Argentina

## Abstract

The structural and mechanical response of metals is intimately connected to phase transformations. For instance, the product of a phase transformation (martensite) is responsible for the extraordinary range of strength and toughness of steel, making it a versatile and important structural material. Although abundant in metals and alloys, the discovery of new phase transformations is not currently a common event and often requires a mix of experimentation, predictive computations, and luck. High-energy pulsed lasers enable the exploration of extreme pressures and temperatures, where such discoveries may lie. The formation of a hexagonal (omega) phase was observed in recovered monocrystalline body-centered cubic tantalum of four crystallographic orientations subjected to an extreme regime of pressure, temperature, and strain-rate. This was accomplished using high-energy pulsed lasers. The omega phase and twinning were identified by transmission electron microscopy at 70 GPa (determined by a corresponding VISAR experiment). It is proposed that the shear stresses generated by the uniaxial strain state of shock compression play an essential role in the transformation. Molecular dynamics simulations show the transformation of small nodules from body-centered cubic to a hexagonal close-packed structure under the same stress state (pressure and shear).

Phase transitions are of utmost importance in determining and controlling the properties of materials. Tantalum is a model body-centered cubic (BCC) metal; its high phase stability with increasing pressure and temperature^1^ has enabled researchers to explore plasticity without complications involved with phase changes.

Yet, there is ongoing experimental and theoretical contention regarding high-pressure, high-temperature polymorphism in tantalum. Burakovsky *et al.*^2^ conducted *ab initio* simulations that predicted the existence of an omega (ω) phase in the high pressure-temperature regime of monocrystalline Ta (above ~70 GPa). Using a density functional theory based model and generalized pseudopotential, Haskins *et al.*^3^ identified a size effect for the hexagonal phase. Shang *et al.*^4^ systematically calculated phase transition pressures for 76 elemental solids including pure tantalum, mentioning a fcc-hcp transition at 67.5 and 285 GPa by using a projector-augmented wave method within a generalized gradient approximation. Experimentally, Hsiung and Lassila^5^,^6^,^7^,^8^ observed twins and the ω phase in pure Ta and a Ta-W alloy. The bcc-to-hexagonal phase transition occurred in the Ta-10 W alloy at about 30 GPa and in polycrystalline Ta at 45 GPa with a 1.8 μs load duration. Hsiung and Lassila^7^,^8^ proposed a mechanism, indicating that shock pressure leads to shear-based transformation in tantalum from the bcc to the ω phase. The possible existence of the ω phase has been discussed extensively in the literature and there is a considerable degree of uncertainty as to its formation and stability relating to impurities and grain boundary effects.

The objective of this report is to describe observations of a solid-solid phase transformation in monocrystalline tantalum with different orientations ([001], [110], [111], [123]), shock compressed at very short durations (~3 ns) and high strain rate (~10^8^ s^−1^) in a uniaxial strain state. These observations are backed by molecular dynamics simulations, making this a powerful case for a phase transition, nucleated in the extreme regime of high pressure, shear strain, and strain-rate generated by high energy pulsed laser compression. The extreme stress state was created by six simultaneous incident laser pulses generating a pressure wave that penetrated into a capsule where a tantalum specimen was placed (Fig. 1); details are provided in the Methods Section.

## BCC-ω Phase Transformation

The transmission electron microscopy (TEM) diffraction patterns for substructures were simulated using the software DIFFRACT. The lattice parameter for BCC Ta is 0.3304 nm; for the twin structures in the BCC Ta matrix, the lattice parameters are the same. The crystallographic relations between the twin structure and matrix can be found in Table S1 within the supplemental material.

In simulating the diffraction patterns of the ω phase, the structure identified by Hsiung and Lassila^7^,^8^ was used. It is a pseudo hexagonal structure with lattice parameters, *a = b* = 0.468 nm *c* = 0.2886 nm, *α = β* = 90°, and *γ* = 120°. The locations of the atoms are (0, 0, 0), (0.6853, 0.3146, 0.5), and (0.3146, 0.6853, 0.5) in unit lattice dimensions. The relations between the ω phase and BCC matrix can be found within the supplemental material. Using a [110] foil normal and [131] zone axis as an example, the extra diffraction spots for twinning and ω in the diffraction pattern are shown in Fig. 2, where their size corresponds to the intensity. The twin plane is 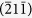, as it is the plane of mirror symmetry between the two pairs of matrix and twin spots, (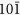) and (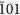), shown in Fig. 2(a). Figure 2(b) is the simulated diffraction pattern with the ω phase substructure spots (hollow circles) in the same matrix and orientation. It is clear that the distribution of extra spots is different from the one for twinning, though there are shared spots as indicated by shaded blue circles. Therefore, the shock-induced substructure can be identified by the electron diffraction pattern (foil normal = [123] and zone axis as [101]) shown in Fig. 2(c). The differences between bcc, twinning, and ω are clear and the structures can be easily identified. Not surprisingly, the spots shared by the ω and twin structures appear the brightest, implying some degree of coexistence.

The substructures observed in our experiment occur after a pulse with of ~3.7 ns duration, 1/500 of that used by Hsiung and Lassila^5^,^6^,^7^,^8^. Figure 3 shows the TEM dark field micrographs for three different orientations: [110], [111], [123]. The corresponding shot energies and foil depth for each orientation are: [110] 625 J at ~200 μm below the bottom of the crater; [111] 661 J at 245 μm below the bottom of the crater, and [123] 633 J at 200 μm below the bottom of the crater. From the LASNEX simulation results, the corresponding pressure at this position is ~71 GPa. The circles in the inserted diffraction patterns indicate the spots used for dark field images. From the simulation software the matrix diffraction pattern can be identified and indicated by zone <131> for [110], zone <113> for [111] and zone <011> for [123] monocrystalline tantalum.

Comparing the morphology of the phase and the diffraction patterns with the results by Hsiung and Lassila^5^,^6^,^7^,^8^, it can be concluded that substructure is the ω phase. The planar boundaries of the ω phase suggest that the interface energy plays an important role; there seems to be a process of minimized energy at specific orientations, a common phenomenon in phase boundaries. The ω domains have a lateral dimension of ~0.2–0.4 μm (Fig. 3(a,b)). These plates are shorter and blockier in [123] monocrystalline Ta (Fig. 3(c)). Hsiung and Lassila^5^,^6^,^7^,^8^ also observed the formation of zigzag-shape ω phase that they suggested was due to the coalescence of omega blocks of the same variant. These faceted grain boundaries are characteristic of massive transformations, in which the boundary moves by a diffusional process, although the composition of product and parent phase are identical. Studied since the fifties (e. g., Massalski^9^), massive transformations can also occur in pure elements and are not restricted to alloys. The facets observed here are very similar to the ones reported by Aaronson^10^.

Electron Back Scattered Diffraction and pole figure analysis of experimental results from Florando *et al.*^11^ indicate that twinning in [110] and [123] oriented monocrystals is relatively more favorable to occur. The volume fraction of ω in these three orientations also can be calculated from the TEM images shown in Fig. 3. The shear (deviatoric) component of stress is significant, and, since the alpha to ω transformation requires a shuffle, it is quite probable that the shear component of stress contributes to the transformation. Shear stresses have been found to have a significant effect on reactions and phase transitions. This was first recognized by Bridgman^12^ and later, by Teller^13^. Enikolopian^14^ systematically investigated the effect in polymers and Chen *et al.*^15^ showed that the threshold pressure for the exothermic reaction between Ti and Si powders was significantly decreased by the superposition of shear stresses. In shock compression, the shear component is very significant and one has the following ratio between shear stress (*τ*_*max*_) and pressure (*p*):


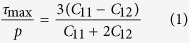


For tantalum, zero pressure *C*_*11*_ = 260.9 GPa and *C*_*12*_ = 165.2 GPa (Sang *et al.*^4^) produce a ratio is 0.49. As plastic deformation and/or shear transformation take place, this value is relaxed. This value will also exhibit a pressure dependence.

The relationship between phase transformation fraction and crystal orientation can be rationalized in terms of the shear stress acting on the planes ({112}) and directions ([111]) of the shuffles involved in the formation of the ω phase. The shear stress can be calculated using the procedure that was outlined by Lu^16^. The state of uniaxial strain in shock compression is represented by the tensor containing only one non-zero component:


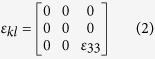


The stresses can be calculated from the strains through the generalized Hooke’s law:


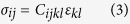


where *C*_*ijkl*_ is the elastic stiffness matrix. This matrix is referenced to an orthogonal frame with axes [100], [010], and [001]. In order to obtain the resolved shear stresses for the other shock compression orientations, the stiffness tensor has to be transformed to the respective coordinate system according to the transformation law below, in which the *l*_*ij*_ are the direction cosines relating the new to the old coordinate system.


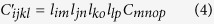


The transformation fractions measured from TEM micrographs for the four shock compression directions are, in descending order: [110]:0.59; [123]:0.34; [111]:0.24; and [001]: <0.05. The latter transformation fraction was extremely low and localized. The maximum resolved shear stress on the most properly oriented {112}[111] system are: [123]:68.1; [110]:63.9; [110]:51.7, and [001]:50. The units of stress are ε_33_GPa and therefore, for each pressure, the corresponding strain has to be calculated from the Rankine-Hugoniot relationships and then inserted into the expression. The [123] orientation has the highest shear stress, 0.69 ε_33_ GPa and corresponds to the second highest transformed fraction, 0.34, whereas [001] has the lowest resolved shear stress, 0.50, and exhibits only a small fraction of the phase transformation that could not be quantified in a previous study^17^. Thus, the shear stress in {112}[111] system seems to be playing an important role. The transformation was also observed in polycrystals with micrometer grain sizes^5^,^6^,^7^,^8^, but not in nanocrystals^18^ indicating that grain-size dependent deformation mechanisms might also play a strong role. However, at this point, no one-to-one correspondences could be found.

## Molecular Dynamics Simulations

Molecular dynamics simulations of shocked [110] tantalum single crystals also revealed a bcc-hexagonal phase transformation above a threshold pressure of 75 GPa and corresponding shear stress of 13 GPa. Simulations used a recently developed EAM potential for Ta^19^. Interestingly, the potential was developed to extend applicability to high pressure with no solid-solid phase transitions, explicitly showing that the bcc-hcp enthalpy barrier is negative up to 460 GPa for an applied hydrostatic pressure^20^. Other work using this potential has not revealed such a phase change even along preferential directions^21^,^22^,^23^. This is consistent with DFT simulations, which show no free-energy crossings as hydrostatic pressure is increased^2^,^24^.

A significant volume fraction of hexagonal clusters immediately following the shock front can be seen in Fig. 4(a). The clusters commonly nucleate near twin boundaries and appear to have high prevalence at twin-twin intersections. They vary in size from 10 atoms to 500 atoms, reaching a few nanometers in diameter. An example of a large cluster can be seen in Fig. 4(b,c) showing van der Waals atomic radius packing and hexagonal bonding respectively. The identification of the hexagonal structure was enabled by adaptive common neighbor analysis^25^, a methodology suited to distinguish components of multi-phase systems through an iteratively defined cutoff parameter. Figure 4(d) illustrates an orientation imaging map of the shock. The original crystal orientation, [110], is colored green and twins are colored red, near <100> orientations. The shock front contains a higher number of twins and a greater volume of the hexagonal phase as compared to the material further behind the shock front. It can be speculated that detwinning and shock induced dislocations might have a significant role in the stability and remaining volume fraction of the hexagonal phase.

## Conclusions

1. We report the observation of an omega (hexagonal) phase transformation in body centered tantalum above a threshold pressure of 70 GPa within 3.7 ns. The transformation pressure is near a factor of two higher and the time is a factor of 500 lower than previous observations by Hsiung and Lassila^5^,^6^,^7^,^8^, (45 GPa at t_p_ ~1.8 μs). The relationship between shock time and transformation pressure is analogous to kinetic constrains for the bcc-fcc phase change in iron, that shifts from 13 GPa to 20–35 GPa at higher strain-rates^26^,^27^,^28^,^29^.
2. In contrast with the current results, calculations for hydrostatic pressure show that the difference in energy between the ω and the β (BCC) phases increases with pressure^2^,^24^. This points to the essential role of shear stresses associated with pressure in the transformation.
3. Molecular dynamics simulations reveal the formation of nanometer hexagonal transformation nodules that are accompanied by significant shear deformation in the lattice. The importance of shear stresses is corroborated in the MD simulations where hydrostatic compression alone cannot produce the transformation.

## Methods

**Figure 1:**
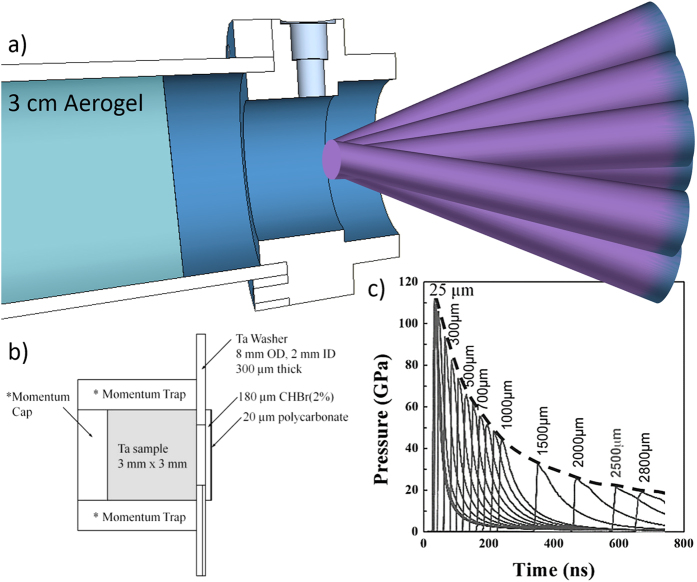
(**a,b**) Assembly used to shock compress the Ta monocrystals; notice momentum trap; (**c**) calculated pressure (using 1-D LASNEX and normalization parameter) as a function of depth for laser shock energy of 684 J.

**Figure 2:**
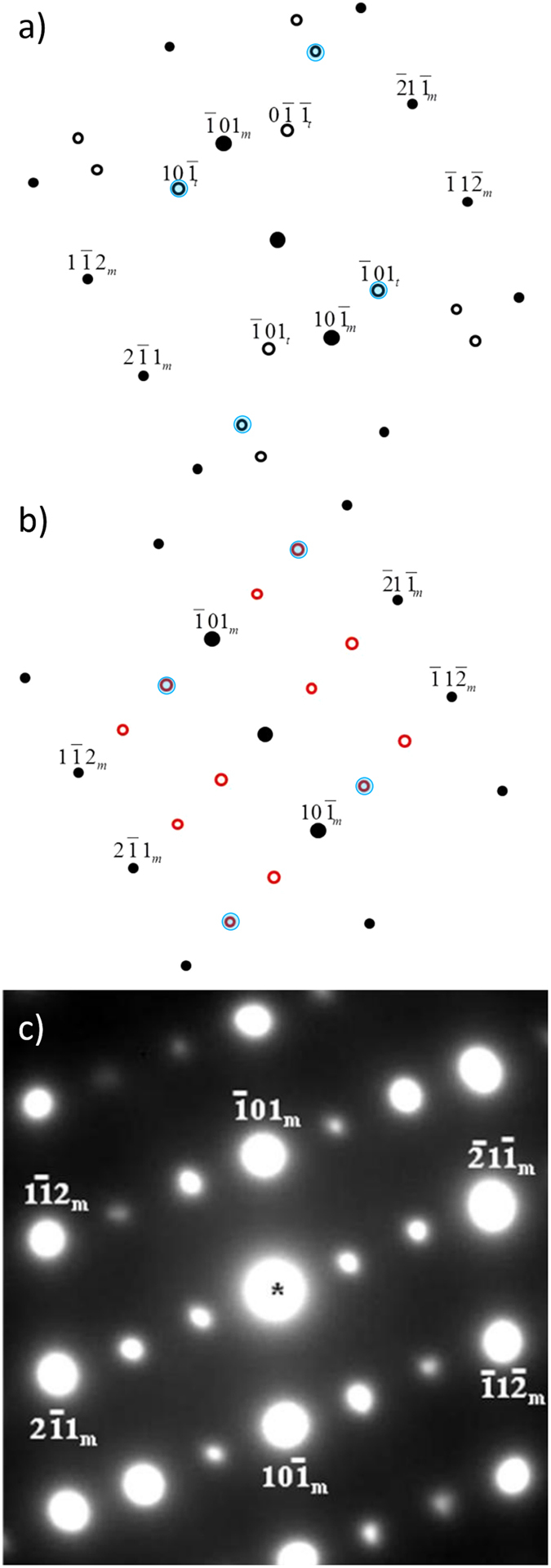
Foil normal [110] and zone axis [131] diffraction patterns. (**a**) Simulated twin spots (hollow circles and subscript t, (**b**) Simulated omega phase spots (red hollow circles). Larger blue circles correspond to shared diffraction spots. (**c**) Experimental diffraction pattern.

**Figure 3:**
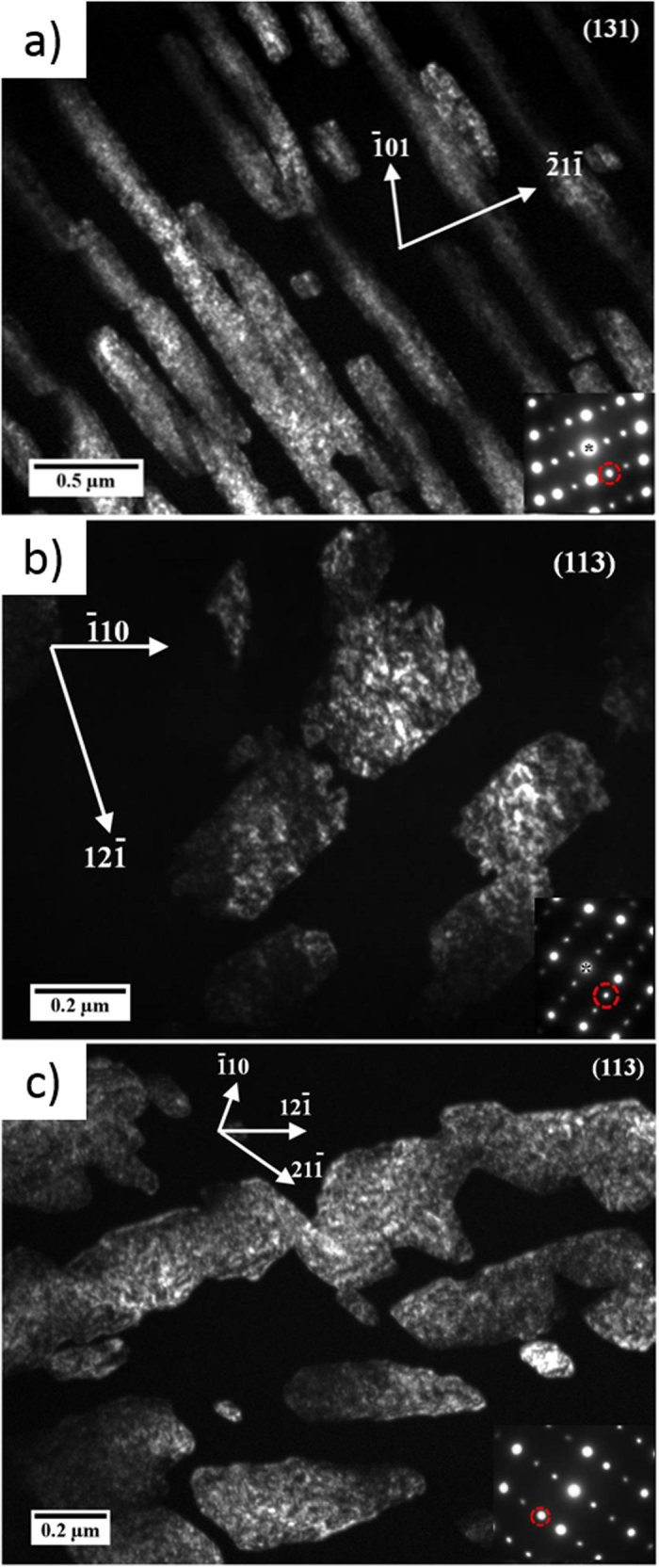
Dark-field TEM images showing ω phase (bright areas) in (a) [110] monocrystalline Ta driven energy of 625 J (foil at 200 μm below the crater, corresponding to pressure of ~71 GPa); (b) [111] monocrystalline Ta driven energy of 661 J (foil at 245 μm below the crater, corresponding to pressure of ~71 GPa); (c) [123] monocrystalline Ta driven energy of 633 J. For a nominal 650 J shot at 200 μm below the crater the corresponding pressure is ~71 GPa.

**Figure 4:**
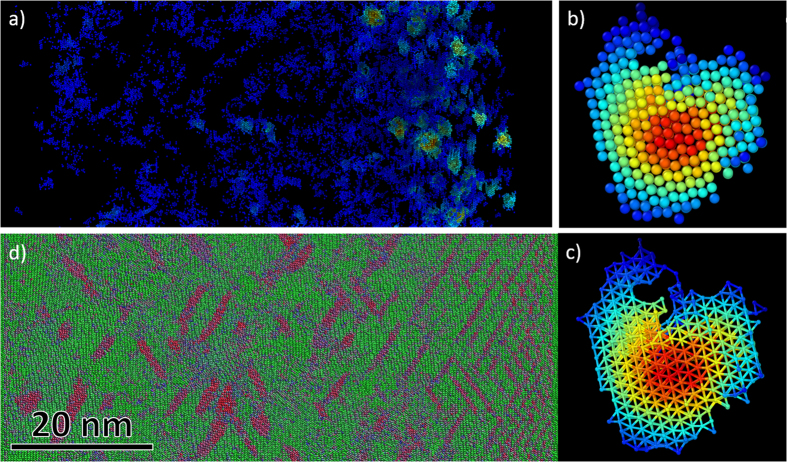
Molecular dynamics simulation of shocked [110] tantalum crystal at a particle velocity of 1.1 km/s (shock pressure near 120 GPa). (**a**) Hexagonal phase as filtered by adaptive common neighbor analysis^25^ and colored by neighbor count. Close-up images of a hexagonal cluster consisting of nearly 500 atoms showing (**b**) packing and (**c**) bonding (imaging by Ovito^35^). (**d**) Orientation imaging map where green corresponds to the <110> direction and red to the <100> direction (imaging through MD_render^36^ implemented in SPaSM^37^).

Pure monocrystalline tantalum of four orientations, [001], [110], [111], and [123], was obtained from MarkeTech Intl, Inc. in cylindrical specimens with dimensions of 3 mm diameter and 3 mm height. The interstitial impurities (ppm in weight) measured by Evans Analytical Group in Ta are O: <10, N: <10, H:7.6 and C: <10. The laser recovery experiments were performed at the Laboratory for Laser Energetics, University of Rochester (Omega Facility). The experimental set-up had previously been tested for recovery of face-centered cubic metals^30^,^31^,^32^,^33^,^29^ and is described in detail elsewhere^16^,^17^,^18^. The target package consists of capsule filled with silica aerogel which acts as a deceleration medium for the tantalum target package after laser shock compression (Fig. 1(a)). The cylindrical tantalum monocrystal targets, as detailed in Fig. 1b, were placed behind a tantalum washer and were backed up by a Ta momentum trap to minimize reflected tensile waves. VISAR (velocity interferometer system for any reflector) experiments of the drive were conducted on calibration samples of Al-LiF. Interface velocity data allowed pressure versus time of varying loading conditions to be deduced, serving as subsequent input in hydrodynamic simulations using 1-D LASNEX. Based on the VISAR data and hydrodynamic calculations, the pressure decay profile as a function of energy can be established with considerable certainty. For an input energy of 684 J, the predicted decay is shown in Fig. 1(c).

The shocked targets were examined using a scanning electron microscope (Phillips XL30 ESEM) and transmission electron microscope (Tecnai F20, operated at 200 kV). Transmission electron microscopy (TEM) foils were mainly prepared by focused ion beam (FIB; Hitach NB-5000 FIB-SEM) techniques due to difficulties in electropolishing the following three orientations: [110], [111], and [123]. The samples prepared for FIB were cut into half and mounted into epoxy. The cross-sections were mechanically polished using Al_2_O_3_ paste down to 0.05 μm and coated with a thin layer of Ir for the FIB milling procedure. The FIB samples were perpendicular to the shock propagation direction, parallel to the surface of the Ta specimens, and had a thickness of 50–100 nm. There are two benefits from this cutting procedure and orientation: (1) the cut samples have a known orientation; (2) from VISAR and computational results, the pressure can be calculated as a function of depth and thus can be established accurately at the foil plane. The pressure decay is assumed independent of orientation. Therefore, for the [110] monocrystalline Ta sample subjected to a 625 J laser-induced shock pulse, the pressure at a distance 200 μm below the crater (crater depth is about 166 μm) is about 71 GPa.

Molecular dynamics simulations were carried out with the LAMMPS package^34^ with the shock wave driven by a frozen piston.

## Additional Information

**How to cite this article**: Lu, C.-H. *et al.* Phase Transformation in Tantalum under Extreme Laser Deformation. *Sci. Rep.* **5**, 15064; doi: 10.1038/srep15064 (2015).

## Supplementary Material

Supplementary Information
