# Supplementary material for: Phase Transformation in Tantalum under Extreme Laser Deformation
Source: Sci Rep. 2015 Oct 19;5:15064. doi: 10.1038/srep15064 (PMC4609924; doi:10.1038/srep15064)
Supplement: Supplementary Information [file srep15064-s1.doc]

**Phase Transformation in Tantalum under Extreme Laser Deformation**

C.-H. Lu, E. N. Hahn, B. A. Remington, B. R. Maddox, E. M. Bringa, M. A. Meyers

**Supplemental Information**

Here we provide orientation relations relevant to bcc-twin and bcc-omega diffraction identification. The “plane” indicates the twin/phase plane while the “direction” indicates the relation between the twin/omega structure and matrix.

Supplemental Table 1: Parameter relationships for twin structure used in DIFFRACT software.

| Plane | Direction |
| --- | --- |
| $\left(21\bar{1}\right)()_{m}$//$\left(21\bar{1}\right)()_{t}$ | $\left[1\bar{1}1\right]_{m}//\left[\bar{1}1\bar{1}\right]_{t}$ |
| // | $\left[11\bar{1}\right]_{m}//\left[\bar{1}\bar{1}1\right]_{t}$ |
| // | $\left[111\right]_{m}//\left[\bar{1}\bar{1}\bar{1}\right]_{t}$ |
| $\left(211\right)_{m}$//$\left(211\right)_{t}$ | $\left[\bar{1}11\right]_{m}//\left[1\bar{1}\bar{1}\right]_{t}$ |
| // | $\left[\bar{1}11\right]_{m}//\left[1\bar{1}\bar{1}\right]_{t}$ |
| // | $\left[111\right]_{m}//\left[\bar{1}\bar{1}\bar{1}\right]_{t}$ |
| // | $\left[1\bar{1}1\right]_{m}//\left[\bar{1}1\bar{1}\right]_{t}$ |
| $\left(112\right)_{m}$//$\left(112\right)_{t}$ | $\left[11\bar{1}\right]_{m}//\left[\bar{1}\bar{1}1\right]_{t}$ |
| $\left(121\right)_{m}$//$\left(121\right)_{t}$ | $\left[1\bar{1}1\right]_{m}//\left[\bar{1}1\bar{1}\right]_{t}$ |
| // | $\left[11\bar{1}\right]_{m}//\left[\bar{1}\bar{1}1\right]_{t}$ |
| //$\left(\bar{1}2\bar{1}\right)_{t}$ | $\left[111\right]_{m}//\left[\bar{1}\bar{1}\bar{1}\right]_{t}$ |
| $\left(12\bar{1}\right)()_{m}$//$\left(12\bar{1}\right)_{t}$ | $\left[\bar{1}11\right]_{m}//\left[1\bar{1}\bar{1}\right]_{t}$ |

Supplemental Table 2: Parameters relationships for Omega phase used in DIFFRACT software.

| Plane | Direction |
| --- | --- |
| $\left(111\right)_{m}//\left(001\right)_{\omega}$ | $\left[1\bar{1}0\right]_{m}//\left[100\right]_{\omega}$ |
| $\left(111\right)_{m}//\left(001\right)_{\omega}$ | $\left[01\bar{1}\right]_{m}//\left[010\right]_{\omega}$ |
| $\left(111\right)_{m}//\left(001\right)_{\omega}$ | $\left[10\bar{1}\right]_{m}//\left[110\right]_{\omega}$ |
|  | $\left[0\bar{1}\bar{1}\right]_{m}//\left[\bar{1}\bar{1}0\right]_{\omega}$ |
| $\left(200\right)_{m}//\left(02\bar{1}\right)()_{\omega}$ | $\left[012\right]_{m}//\left[\bar{1}\bar{1}2\right]_{\omega}$ |
|  | $\left[311\right]_{m}//\left[\bar{1}\bar{1}\bar{1}\right]_{\omega}$ |
